# Politics of hesitance and the formation of ethical subjects through responsible gambling practices

**DOI:** 10.3389/fsoc.2022.1032752

**Published:** 2022-12-14

**Authors:** Jani Selin

**Affiliations:** Health and Well-Being Promotion, Department of Public Health and Welfare, Finnish Institute for Health and Welfare, Helsinki, Finland

**Keywords:** gambling, governmentality, Foucault (Michel), subject formation, ethics

## Abstract

**Introduction:**

Instead of harm prevention and risk related to gambling products, responsible gambling discourse emphasizes the importance of voluntary measures. From the point of view of governmentality, the responsible gambling practices produce, rely on, and call forth subjectivities. The aim of the study is to find out what kind of subjects are being produced through the responsible gambling practices of four Nordic state-owned gambling companies. As the companies are state-owned and operate on different types of markets, there are reasons to suspect that the companies could endorse different versions of the figure of responsible gambler. Previous research indicates that responsible gambling practices typically presuppose or aim to produce a self-governing subject making informed choices. Less attention has been given to detailed analyses of the heterogeneous factors contributing to the ethical subject formation. Moreover, there is a growing body of literature indicating, that along with the use of detailed behavioral data (big data), new forms of governmentality, that are highly relevant from the point of view of subject formation, are emerging.

**Methods:**

The responsible gambling practices are analyzed along Michel Foucault's four aspects of ethics. First, there is ethical substance, the problematic aspect of the self that is taken as the target of the ethical work. Second, the subject needs to have a certain relation to social norms and moral codes. Third, ethical work is needed to mold the problematic aspect of the self. Fourth, the aim of the ethical work is a certain mode of being or relationship to oneself. The analyzed material consists of the annual reports of the companies between 2019 and 2021.

**Results and discussion:**

The results show that the figure of subject making informed voluntary choices is deeply embedded in the responsible gambling practices of the companies. The companies entice the gamblers to think about themselves and to act upon themselves as subjects capable of self-control. Hesitancy to intervene characterizes the activities of the companies, even if all the companies collect and use detailed behavioral data. The inclusion of the precarious subject of gambling harm would allow the companies to do much more to prevent gambling harm.

## 1. Introduction

In this article I analyze what kind of subjects are being produced through the responsible gambling practices of four Nordic state-owned gambling companies. Responsible gambling discourse with its emphasis on individual responsibility is widely endorsed by the gambling industry (Alexius, 2017; Hancock and Smith, 2017). Instead of harm prevention and risk related to gambling products, responsible gambling discourse accentuates the importance of education and voluntary responsible gambling tools that are supposed to help gamblers to control their gambling behavior (Livingstone and Rintoul, 2020). Moreover, typically responsible gambling discourse tends to represent gambling as fun and leisure, and the problems related to it as an issue only for a minority (Francis and Livingstone, 2021).

The responsibilization of gamblers relates to the more general development in governmentality, addressed in vast critical sociological, public health and addictions literature (Lupton, 1995; Petersen and Bunton, 1997; Rose, 1999; Moore and Fraser, 2006; Reith, 2007), by which power is exercised more and more by relying on the voluntary choices of the calculating economic subjects. There is a tendency in neoliberal governmentality to focus on creating conditions under which economic subjects can make rational decisions (Rose, 1999; Foucault, 2008). However, the individuals and groups who do not or cannot submit to neoliberal government can be categorized as pathological or abnormal and can be subject to normalizing power (Reith, 2007). The critical literature on public health shows that new public health relies on responsibilization of individuals and their self-control, but this does not mark the disappearance of disciplinary power (Lupton, 1995; Petersen and Bunton, 1997). Rather, assemblages of government, self-government and knowledge are to be understood as heterogeneous, consisting of different technologies of power and producing and relying on a variety of subjectivities (Foucault, 1978, 2007; O'Malley, 1996; Collier, 2009).

Governmentality studies have been criticized for being too totalizing and not paying enough attention to the exact compositions of the assemblages that “*cannot* be made intelligible through reference to common conditions of possibility” (Collier, 2009, p. 98, emphasis original), such as “neoliberal governmentality” or “disciplinary power”. There is thus need for detailed empirical studies.

Foucauldian analyses of the discourses and practices of responsible gambling have shown how historically and institutionally the categories of the responsible gambler, a subject making rational informed choices, and the problem gambler, a subject that consumes itself through consumption, are tied to the rise of gambling the industry and neoliberal governmentality (Reith, 2004, 2007, 2008; Young, 2013). Reith (2007) analyses in detail the genealogy of pathological gambler and shows how different subject positions have been produced in the expert discourses on problem gambling: the malleable subject of treatment, the risky subject of public health, the dependent subject of physiology, the irrational subject with distorted cognitions, and the uncontrolled subject of craving or addiction.

However, there are also studies in which the “totalizing logic of biopolitical government” (Collier, 2009, p. 80) is clearly foregrounded. Miller et al. (2016), apply the governmentality approach to the analysis of the responsible gambling discourses of the gambling industry and the government in Australia. They show how the figures of the responsible gambler and the problem gambler are discursively produced, and suggest that gamblers are through disciplinary surveillance enticed to monitor their gambling behavior. Allsopp (2021, p. 57) also sees the use of behavioral data of gambling companies as a method for the surveillance and discipline of subjects who “are ultimately expected to internalize and regulate their own behavior”.

Foucault (1997a, p. 225) defines governmentality as the “encounter between the technologies of domination of others and those of the self”. However, there is paucity of detailed empirical studies on ethical subject formation through technologies or practices of the self (Foucault, 1985) in sociological gambling research. Schüll's (2006) ethnography of people recovering people from problem gambling in Las Vegas is exceptional as its focus is on subject formation.

Sociologically Schüll contributes to literature on the formation of neoliberal subjects, understood as idealized utility-maximizing entrepreneurs of the self (Rose, 1996, 1999; Foucault, 2008; Lindner, 2020; Van Wijk, 2021). Schüll shows how the ethical practice of the self of recovering gamblers, living in an environment where the gambling industry and the addiction recovery industry are present, is not typically “hinged to any particular ethical telos, but rather carries over from the domain of self-recovery to the domain of self-loss, and back again” (Schüll's, 2006, p. 233). Schüll's (2006, p. 241) contribution shows that there are “aspects of normative neoliberal subjectivity that analyses framed solely on models of entrepreneurial selfhood miss”.

Schüll's insights are still topical to debates on the formation of neoliberal subjectivity (Lupton, 2012; Christiaens, 2020; Lindner, 2020). Lindner (2020, p. 88) argues that there is at least a partial break between neoliberal government with its emphasis on individual choice and new forms of government related to digital applications and games that rely “on the non-rational, emotionally-acting *homo ludens*”, contributing to “ethical formation of the self, based on sensor-generated body-behavior data”. Lupton (2012), analyzing mobile health promotion applications, also emphasizes that subject formation consists of heterogeneous elements, both disciplinary and neoliberal. Barry (2019, p. 365-366) also underlines that digital governmentality employing big data differs from disciplinary and neoliberal governmentality because expert knowledge is largely abandoned and government is exercised through “incitation and manipulation” (see also Newall, 2019). Following these lines of interpretation, in this paper the constellations of governmental and ethical practices of subject formation are understood and approached as temporally and spatially heterogeneous.

The aim is to analyze in detail what kind of subjects are produced, relied on, and called forth through the heterogeneous practices of responsible gambling. The focus of the article is on the intersection of subject formation and new forms of governmentality, the digital governmentality employing big data in particular. How are the gamblers led to recognize themselves as certain kinds of subjects responsible for their gambling behavior? Are there discernible differences between the companies from the perspective of subject formation?

## 2. Responsible gambling, governmentality, and ethical subject formation

Responsible gambling has been characterized as one strategy of the gambling industry to shape public health policy in order to protect business interests (Selin, 2016; Knai et al., 2021). Many of the critics of responsible gambling represent public health as an alternative approach (Livingstone and Rintoul, 2020; Van Schalkwyk et al., 2021). What differentiates the public health approach from the responsible gambling is its emphasis on preventive measures targeting the whole population, such as restrictions on the availability of gambling and the structural features of the most harmful gambling products (Livingstone and Rintoul, 2020, p. 3). However, it needs to be noted that responsible gambling and public health have commonalities. Responsible gambling can be considered as a form of educational health promotion (Price et al., 2021). Furthermore, the justification of both responsible gambling and public health interventions stems typically from their alleged ability to prevent or reduce gambling harm. From the point of view of governmentality there are no clear normative standards, such as gambling harm, to value different ways of governing human behavior (Tiisala, 2017; Rae, 2022). Therefore, both responsible gambling and public health practices are to be considered here as government and are analyzed from the perspective of subject production or subject formation.

Government and governmentality had a key place in Foucault's thought in the 1970's but in the 1980's Foucault became more preoccupied with the link between the government of others and self-government of subjects (Foucault, 1985, 1997a,b). The subject positions in discourse, ethical self-government and the production of subjects through the technologies of power offer each different perspectives to subject formation (Foucault, 1985).

In Foucault's late thought government refers to power relations in general, to practices that seek to modify the actions of subjects insofar as the subjects are free to resist the power (Foucault, 1983, 2008). By governmentality Foucault (2007) refers to the historically specific and contingent rationalities and practices used to organize the government of individuals and collectives. Foucault first analyzed governmentality organized around normalization and usually employing knowledge produced by the human sciences (Foucault, 1978, 1979). The main objects of this modern way of exercising power were the population and the individual (Foucault, 1978, 2007). Normalization constitutes subjects by shaping their experiences of the world and of themselves; it entices individuals to seek “the truth about one's normality and to correct potential deviations from the norm” (Tiisala, 2021, p. 28). Modern government is in this way connected to the practices of self-government that allow individuals to “effect by their own means, or with the help of others, a certain number of operations on their own bodies and souls, thoughts, conduct, and way of being” (Foucault, 1997a, p. 225).

Self-government of subjects was a crucial aspect in the way Foucault understood ethics. For Foucault morality consisted of codes of conduct and actual moral behavior, whereas ethics is “the manner in which one ought to form oneself as an ethical subject acting in reference to the prescriptive elements that make up the [moral] code” (Foucault, 1985, p. 26). In the context of sexuality, ethics was closely connected to power, to the ways individuals are “led to focus their attention on themselves, to decipher, recognize, and acknowledge themselves as subjects of desire” (Foucault, 1985, p. 5; see Foucault, 2021, p. 285).

Foucault (1985, p. 26–28) analyzed ethics along four dimension or aspects. First, there is the *ethical substance*, the constitution of a part of the self as the “prime material” of moral care. The ethical substance can for example consist of strict rule-following behavior, or mastery of thoughts, emotions, and urges or desires related to gambling. Second, *the mode of subjection* characterizes the way and reasons why the individual acts according to a moral code. Custom or tradition can be the reason for rule-following, but it is possible that the individual wishes to communicate to others one's commitment to certain standards of behavior. Third, *ethical work*, consists of becoming an ethical subject, for example, through different exercises of abstinence, or practices or techniques of interpretation and monitoring of one's emotions and thoughts (see Selin, 2011). Finally, there is the *telos* of the ethical subject, the mode of being that the individual considers characteristic of an ethical subject. This *telos* can for example be full self-control, abstinence, or the ability to gamble moderately on every imaginable occasion. These four aspects of ethics guide the analysis of the empirical material.

Analysis of subject formation along Foucault (1985) four aspects of ethics (mode of subjection, techniques of the self, ethical substance, telos) offers a refined way to empirically study subject formation through the practices of responsible gambling without being committed, for example, to too general a conception of a neoliberal subject or neoliberal government and contributing thus to better understanding of the power relations embedded in the responsible gambling practices of the gambling industry. Moreover, the gathering and employment of behavioral data by gambling companies underline that just like other digital data companies, gambling companies have an interest in using the data to monitor and to modulate the behavior of gamblers in profit-optimizing ways (Allsopp, 2021). Empirical studies of subject formation in the context of gambling can contribute to better sociological understanding of the emerging political and ethical challenges related both to the formation of subjects and to the use of big data (see Rouvroy and Berns, 2013).

Through this paper, the responsible gambling practices of four Nordic state-owned gambling companies (Danske Spil, Norsk Tipping, Svenska Spel, Veikkaus) are analyzed as subject formation. The status of the companies as state-owned makes them an interesting object of study because all the companies claim to be particularly responsible and underline that they contribute their revenue to different societal purposes (Cisneros Örnberg and Tammi, 2011; Selin, 2016; Alexius, 2017). The companies are also a prime example of the conflict of interest the governments have as the beneficiaries of gambling revenue and the preventers of gambling harm (Sulkunen et al., 2018). It is thus possible that the companies are more often pushed into taking gambling harm more seriously than their privately owned counterparts. Moreover, all the state-owned companies also request mandatory player identification and collect detailed information on the gambling behavior of their customers. This offers a unique chance to consider the use of this kind of data from the perspective of subject formation. The major difference between the companies is that the companies operate under different regulatory frameworks: Svenska Spel in Sweden and Danske Spil in Denmark operate online under license-based regulatory frameworks with dozens of other legally operating companies. Norsk Tipping in Norway and Veikkaus in Finland operate under monopolistic regulatory frameworks with a public health justification. The companies also have different product portfolios (Nikkinen and Marionneau, 2021). It is thus possible that the companies could employ different responsible gambling practices.

## 3. Data and method

The data analyzed here consists of the annual reports (*N* = 12) of Danske Spil (2020, 2021, 2022), Norsk Tipping (2020, 2021, 2022), Svenska Spel (2020, 2021, 2022), and Veikkaus (2020, 2021, 2022) between 2019 and 2021. The reports are publicly available at the websites of the companies. There were some differences in the content of the reports. Most notably, the most extensive discussion on responsible gambling was found in the annual reports of Veikkaus.

The reports are mostly directed at regulators, politicians, and other stakeholders with an interest in the companies and their operations. Thus, they are not written to be read and contemplated by the gamblers. Yet they all contain detailed descriptions of the responsible gambling practices and tools the companies present as feasible in preventing and reducing gambling harm. Responsible gambling is understood in this study primarily as measures that the operators characterize as responsible gambling measures. Typically, these measures require active agency and thinking by the gamblers, not just submission to injunctions (e.g., age limits). Injunctions are included in the analysis, if they are explicitly mentioned in the context of responsible gambling. Even though the reports are not written for the gamblers, the responsible gambling practices described in them are nevertheless prescriptive in the sense that they are supposed to be adopted by the gamblers to modify their gambling behavior.

The reports with their detailed descriptions of the responsible gambling practices offer insights into the ways the companies rationalize, problematize and identify the aspects of the behavior of the gamblers they consider problematic, and seek to modify. Moreover, analyzing the responsible gambling practices as subject formation will necessarily disclose something about the ethical subjects that the companies seek to produce. The assumption here is that the companies might not have a very clear idea of what they are doing in terms of subject formation.

In the qualitative analysis of the reports, a basic idea of qualitative research, thematic coding, was employed (Nowell et al., 2017). ATLAS.ti version 9 was used to facilitate the analysis. The author was alone responsible for the analysis. The steps suggested for thematic analysis by Nowell et al. (2017) were applied. First, the texts were read. Second, a primary coding, consisting of all paragraphs where words related to responsible gambling occurred and responsible gambling practices were discussed, was conducted. When identifying the responsible gambling sections of the reports the following base words were used as search terms: “vastuullinen” for the reports written in Finnish and, “ansvar” for reports written in the Scandinavian languages. Third, all irrelevant parts of the texts were excluded. These excluded parts of the reports typically discussed the corporate social responsibility in general, the sharing of responsibilities within the companies, or the auditing process. Fourth, descriptions of the responsible gambling practices to be adopted by the gamblers were identified. Fifth, the descriptions were translated into English and coded deductively along the four dimensions of ethical subject: the mode of subjection, the ethical substance, the ethical work, and the telos. The development of this coding framework was reviewed and repeated until all practices were consistently categorized along the four dimensions. Finally, to validate the results and their presentation, they were interwoven and discussed with relevant literature. Thus, the results are presented and discussed in tandem.

## 4. Responsible gambling practices as subject formation

As explained above, all the responsible gambling practices were analyzed as prescriptions, as interventions seeking to modify the behavior of the gamblers. The results are presented in the following way. The different responsible gambling practices of the gambling companies are presented. After this, each practice is analyzed and discussed along the four dimensions of subject formation.

The Nordic state-owned gambling companies all use quite similar responsible gambling practices. The key practices are presented in Table 1. There were five main categories of responsible gambling practices: communicational practices providing information on responsible gambling and gambling harm, spending limits and self-exclusion options, practices of contacting the gamblers, self-tests offered for the gamblers, and practices based on actual behavioral data.

**Table 1:** Key responsible gambling (RG) practices of the Nordic state-owned gambling companies as reported in the annual reports.

		**Danske spil**	**Norsk tipping**	**Svenska spel**	**Veikkaus**
Messaging	General information on RG	x	x		x
	Information on product risk and addiction	x			x
	Information on RG tools		x		x
	Moderate marketing			x	
	Information on moderate gambling	x	x		
	Information on help				x
Spending limits and	Mandatory self-set spending limits		x	x	x
self-exclusion	Limits on use of time			x	
	Voluntary daily spending limits				x
	Spending limits at casino				x
	Self-exclusion	x	x	x	x
Behavioral screens	Problem gambling tests	x		x	x
Contacting gamblers	Care calls	x	x	x	x
	Discussion with returning gamblers after casino self-exclusion				x
Use of behavioral data	Personalized feedback	x	x	x	
	Personal gambling history available		x		x
	Automated gambler risk estimation	x	x	x	

### 4.1. Civilizing messages

The communicational practices consisted of providing information about available responsible gambling tools and about the importance of playing responsibly: “We regularly send out emails to our customers, where we inform them about tools for responsible gaming and the importance of playing moderately” (Danske Spil, 2020, p. 11, translation by the author). Moreover, there was messaging about gambling harm, product risk, and availability of help and support. In the annual reports of Svenska Spel there were the fewest mentions of communicational practices. Sometimes the messaging was reported taking place in person at a gambling venue.

The mode of subjection in this practice is not based on rule-following or unconditional acceptance of the authority of the company. The mode of subjection is reminiscent of the “empowering” techniques of government that rely on active participation and seek to actualize the potential capabilities of the subject as a citizen (Cruikshank, 1996). This approach has been widely used in health promotion, for example (Lupton, 1995). In empowerment the responsibility of the subject is foregrounded. The ethical substance is typically the internalization of the information provided. The purpose is to equip the individual with the sort of information that she can voluntarily adopt if needed. The content of the ethical work required from the subject is indefinite. There is nothing specific the subject needs to do. The individual is thus free to choose how to act, how to for example internalize the information, to put it to work when gambling. As the content of the information provided varies, so varies the telos. In most cases the purpose of the messages seems to be a subject who is, when equipped with the required information, able to gamble without harming oneself or others.

This suggests that the communicative practices of the gambling companies are anchored to the ideal of the self-controlling subject of the neoliberal practices of government (Reith, 2007). They can be considered being part of *civilizing technologies* the “means of producing states in which individuals are healthier, more responsible and more able to adhere to the duties, expectations and obligations of their families and societies” (Vrecko, 2010, p. 45). However, in this case the exact content of the pursued states and the techniques to be used remain largely undetermined, allowing the subject thus a large degree of freedom in self-fashioning.

### 4.2. Self-limiting subjects

The spending limits and self-exclusion options consist of practices directed at decreasing the money and time the gamblers use. The gamblers must set their own spending limits when gambling at Norsk Tipping, Svenska Spel and Veikkaus. In the annual reports of Danske Spil spending limits were only mentioned in the analyzed texts in the context of an intervention executed when the company has identified problematic gambling behavior and contacts the gambler. Veikkaus (2021) described the different spending limits in the most detailed fashion: “…*player needs to set* daily and monthly deposit limits before it is possible to transfer money to the player account. Playing the fast-speed games on the Veikkaus online platform requires the setting of monthly and daily loss limits” (p. 22, translation by the author, emphasis added). Self-exclusion options were mentioned in the annual reports of all the companies. In Denmark and Sweden, the self-exclusion registers are national and used by all the licensed companies.

Spending limits hinge on and foster the calculative capabilities of the subject. At least to a certain degree, calculation is inscribed in the practices. It is possible to discern a clear ethical telos in the practices: self-control or even abstinence through calculation of costs and benefits of gambling in monetary terms.

The ethical work or the techniques through which the calculation is realized are indefinite. Yet the calculation of losses and time spent can require the adoption of different techniques of planning, such as budgeting, which require reflexivity and awareness.

The ethical substance, the content of the ethical problematization of certain aspects of behavior, is not explicitly mentioned in the descriptions of spending limits or self-exclusion. However, in the history of addiction “desire” or “urge” have been the common notions to refer to compulsive behavior (Valverde, 1998). Self-control can thus be related to self-monitoring or reflexivity or awareness through which the subject tries to identify the different states, feelings, thoughts, or behavioral patterns that might be signs of the imminent urge to gamble.

The mode of subjection is firmly based on the freedom to choose the spending limits or to exclude oneself from gambling. Therefore, even if the gamblers are nudged toward calculation, cost-benefit analyses, and self-reflexivity, in the end it is a personal choice to do so. This is reminiscent of the prudent subjects of risk who are to be responsible, calculating, and self-controlling (O'Malley, 1996). The calculating subject can be seen as “a self that calculates *about* itself and that acts *upon* itself in order to better itself” (Rose, 1996, p. 154, emphasis original).

### 4.3. Representing the self to the self

Behavioral screens or gambling self-tests consist of questions concerning gambling behavior and are typically offered for the gamblers on the websites of the companies. After the result of the test, the gamblers are offered feedback, possibly given a recommendation to change their behavior, or even contacted by the company experts (Svenska Spel offers this options). Danske Spil (2021, p. 12, translation by the author) reports of introducing a refined practice in 2020: “Next year we will…implement an early warning system that must be able to detect in a short time if a player loses control of his game– and thus give us the opportunity to act faster than we do today”.

Danske Spil, Nork Tipping and Svenska Spel use the practice of contacting by phone those gamblers with behaviors indicating difficulties in controlling their gambling. In this practice expert counselors approach gamblers on the basis of actual behavioral data. Veikkaus piloted these care calls in 2020. In Denmark the practice was related to regulatory requirements. Svenska Spel, Norsk Tipping and Danske Spil emphasize the importance of this practice in their reports. Svenska Spel reports that their monitoring algorithm enables automated restrictions on gambling: “The work takes place through the development of digital game control tools such as our real-time interventions, a built-in system in our games, which enables Svenska Spel to stop customers who play too much in real time” (Svenska Spel, 2021, p. 34, translation by the author).

In addition to care calls, Veikkaus reports having a mandatory discussion with customers returning to a casino after a self-exclusion period.

Care calls are closely connected with the practice of using behavioral data as a basis of interventions directed at changing the behavior of the gamblers. Sometimes personalized feedback is given through email or during the gambling session. Svenska Spel and Norsk Tipping report on using the same algorithm to monitor gambling the behavior of the users of their services.

As the spending limits and self-exclusion options are supposed to entice self-control and calculation through self-reflexivity, the self-tests, the care calls, and the use of behavioral data also seek to produce self-controlling subjects without defining the content of the ethical work. But instead of subtly enticed self-reflexivity, the self-tests rely on the representation of the self to the self.

The self-tests contain questions similar to the questions in the screening instruments used in the population studies of problem gambling. The tests offer a quantified representation of the self, indicating the degree of self-control the subject has (Rose, 1996). Upon the result of the self-test the gamblers are given recommendations concerning their gambling and are possibly advised to seek help. In exactly the same way as the gamblers are free to decide on the use the self-test, they are free to decide whether they follow the recommendations. It is thus the responsibility of the subject to decide what to do after the results of a self-test.

The gambling companies collect enormous amounts of behavioral data on the gamblers. Of the companies at least Veikkaus and Norsk Tipping offer this data to their customers in the form of an individual gambling history, representing the gambling behavior of the individual in terms of overall spending and the stakes bet, for example. Norsk Tipping (2021) considers gambling history to be one the benefits of playing as a registered customer: “Through easily accessible information, *players are more able to make informed choices* about their own gambling habits” (p. 13, translation by the author, emphasis added). Here the subject is again left to decide how to respond to the information provided by the company. What is fascinating in this practice is the use of the visualization of the gambling behavior of the individual to entice problematization of some aspects of behavior (Rose, 1996). The visualization also functions as the representation of the pathological or disorderly aspect of the self to the self. The likely ethical telos in this case is the subject's self-control either alone or with the help of others.

The algorithmic identification of the problematic patterns of gambling from the behavioral data is based on the diagnostic criteria of the gambling disorder or other indicators of problem gambling. The figure of problem gambler is thus implied but it is not in the foreground (see Reith, 2007). The responsibilities and capabilities of the subject are emphasized when the practice of giving gamblers behavioral feedback due to their risky gambling behavior is considered. All the companies have hired expert counselors to deliver the behavioral feedback *via* the care calls and to offer their guidance. Still, in the end the gamblers are free to choose their course of action. Danske Spil (2022) says this explicitly: “Our purpose is not to diagnose gambling addiction or to convince the player to act in a certain way” (p. 16, translation by the author). So even though the algorithms the companies use are built on the knowledge of the gambling disorder and are used to categorize the gamblers according to their individual risk-levels (see Allsopp, 2021), the neoliberal subject with its capacity to make informed choices is prioritized, the only exception being Svenska Spel's real-time intervention practice.

The gamblers with difficulties in containing their gambling behavior are merely approached and given the opportunity to *choose* help-seeking or strive for abstinence and self-control. To put differently, all the companies show incredible *hesitancy* to interfere with the gamblers' freedom to choose. Following the logic on neoliberal governmental rationality (Foucault, 2008; Selin, 2016), the companies seek to create conditions under which the subject can make rational decisions. It also seems that the gambling companies are using the data to create conditions that maintain gambling and optimize their flow of revenue (Allsopp, 2021). This follows the logic of digital governmentality according to which recorded traces of behavior are used “to create excitement, curiosity and addiction, that further incites us to leave more digital traces” (Barry, 2019, p. 375).

The findings above need to be contrasted with the findings of Allsopp (2021). When analyzing the gambling legislation in the UK from a Foucauldian perspective, Allsopp (2021, pp. 65–66) sees the requirement to contact the gamblers and to recommend behavioral changes to them as an exercise of disciplinary governmentality. Moreover, Allsopp (2021, p. 73) maintains that the gambling companies use gambling data to create a “responsible' standard of gambling” and “to ensure that gamblers internalize this standard and ultimately transform into self-regulatory individual”. While there is no reason to reject out of hand that the normalizing power exercised by the gambling companies targets both the gambling population and the individual gambler, this seems unlikely. Insofar as the result of discipline is the permanent internalization of a norm through continuous surveillance and behavioral correction (Foucault, 1979), it is difficult to see why the gambling operators, of whose revenue as much as 60% is attributable to those experiencing harm (The House of Lords, 2020), would promote permanent behavioral changes, resulting in a significant decrease in revenue. As Allsopp also points out, the neoliberal turn in governmentality with an emphasis on individual freedom and responsibility relies on the creation of suitable conditions for the exercise of freedom, not primarily on discipline and normalization (Foucault, 2008).

Finally, there is an interesting link with the use of the gambling data by the gambling companies and the data produced by self-tracking devices and applications. According to Lupton (2012) modern health promotion technologies produce subjects willing to open themselves to continuous measurement and surveillance, and to care for themselves by following health guidance provided by the technologies. Lindner (2020, p. 87) argues that gaming and gambling have served as models for some self-tracking technologies because they produce “an attitude of playfulness in which non-reflexive interaction with the gadget becomes a purpose in itself”. This observation alludes to a fundamental difference between gambling companies and companies selling self-tracking technologies. They both, of course, use the data to hold on to the users of their services. However, the self-tracking technologies provide the users with continuous flow of health-related data to be shared with other users, and to be used in ethical self-formation, whereas the gambling companies actively provide personalized information on gambling harm only to those gamblers with difficulties in controlling their gambling. Paradoxically, these difficulties are likely to be a result of the use of behavioral data in designing and operating the gambling products.

In summary, the findings show that the companies use civilizing *messages* to offer information on gambling harm to gamblers while leaving to gamblers to choose how to put the information to work. The ethical telos is to be able to gamble without harming oneself. Spending limits hinge on and foster the calculative capabilities of the self-limiting subject. There is also a clear ethical telos: self-controlling subject who can recognize any internal sign of the urge to gamble. Self-tests, gambling history and care calls are ways of representing the self to the self. They offer quantified, visualized, or verbalized representation of the gambling self. The gamblers can strive for self-control if they want to. The mode of subjection in all the practices is the freedom to choose. Quite surprisingly, all the companies, despite their differences in terms of the products they sell and the legal frameworks under which they operate (Nikkinen and Marionneau, 2021), seem to endorse the neoliberal version of the subject.

## 5. Conclusion

The purpose of this study was to offer a detailed empirical analysis of the assemblage of responsible gambling practices from the point of view of ethical subject formation and new forms of (digital) governmentality. The need for detailed empirical analyses has been suggested as one possible correction for the problem of making too totalizing interpretations in governmentality studies (Collier, 2009). Analysis of subject formation along the four dimensions of Foucault's ethics is one possible way of analyzing heterogeneous practices in more detailed a fashion. There is paucity of detailed empirical studies on ethical subject formation in sociological gambling research. Moreover, only by paying attention to the details it is possible to discern new emerging forms of digital government related to big data.

From the point of view of subject formation, the differences between the responsible gambling practices of the Nordic state-owned gambling companies were modest. The main finding of the analysis of the self-formation of the subjects is that all the gambling companies share a hesitancy to intervene in the behavior of the gamblers. This finding is consistent with the previous governmentality literature where the avoidance governing too much is identified as characteristic of both liberal and neoliberal governmentality (O'Malley, 1996).

As shown in previous research in other contexts, there is also a discernible tendency to emphasize the responsibility of the gamblers themselves (Young, 2013; Hancock and Smith, 2017). Yet, the companies at the same time collect minute behavioral data on gamblers and use the data to identify risky patterns of play. They have even used the data to develop interventions to contact the gamblers and encourage them to change their behavior. Following the logic of digital governmentality, the companies are likely to use the same or similar data to develop and to promote new products. Despite all this, when it comes to prevention of gambling harm the hesitancy to interfere prevails. The gambling companies rather try to modify the behavior of the people experiencing harm than the harmful features of their products. This indicates how deeply embedded the ideal of a neoliberal subject making rational informed choices is inscribed in the responsible gambling practices of the state-owned Nordic gambling companies: the companies entice the gamblers to think about themselves and to act upon themselves as calculating subjects capable of self-control. Fostering such capabilities equals to harm prevention. This is truly remarkable considering the societal purposes of wellbeing and welfare the companies like to link themselves with. The inclusion of the precarious subject of gambling harm in interventions and in the designing of the gambling products would allow the companies to do much more to prevent gambling harm.

Similar sociological analysis of subject formation could be extended to gambling therapies. Following the work of Rose (1996) and Hacking (2002) it would be possible to investigate the therapeutic rationalities and practices employed in different contexts. Detailed empirical analyses of subject formation would deepen our understanding of the different social forces contributing to the experiences and self-understanding of people experiencing gambling harm. Such research would not only generate knowledge of how the culturally and politically powerful images of being a person with gambling problems are produced and reproduced, but it would also critically engage with the political and economic structures maintaining such images. Moreover, more research is needed on the ethical and political implications and effects of the use of gambling data in gambling operations, the development of gambling products, prevention, and policy. Such research connects the gambling studies firmly to some of the sociologically most interesting questions of the present.

### 5.1. This study has limitations

There is possibility of subjective bias as the qualitative analysis was conducted by a single person. Based on the data used here, nothing can be said of the actual techniques of the self the gamblers employ to alter their gambling behavior. Such analysis would have required other kind of textual data, interviews, or diaries, for instance. Moreover, the focus of the analysis was on the practices that the companies described in their annual reports and usually required the active participation of the gamblers. Thus, some mandatory measures for the prevention of gambling harm, such as age limits, restrictions on marketing, or payment-blocking were outside the scope of this analysis. Their inclusion could have offered insights into the formation of subjects, for example, through the interactions between neoliberal and disciplinary forms of government. However, the annual reports did not contain exhaustive information on the national legal frameworks to facilitate this kind of analysis, and the reports were in themselves such a rich data set that it was sensible to analyze it thoroughly and not to widen the scope of the study in new directions. Furthermore, the inclusion of legislation to the analysis of responsible gambling practices could have problematically conflated responsible gambling with statutory harm prevention.

## Data availability statement

The original contributions presented in the study are included in the article/supplementary material, further inquiries can be directed to the corresponding author.

## Author contributions

JS is the author of the paper. He designed the study, collected, analyzed the data, wrote the manuscript, and approved the submitted version.

## References

[B1] Alexius S. (2017). Assigning responsibility for gambling-related harm: scrutinizing processes of direct and indirect consumer responsibilization of gamblers in Sweden. Addict. Res. Theor. 25, 462–475. 10.1080/16066359.2017.1321739

[B2] Allsopp R. (2021). Leveraging the 'power' of big data in the production of 'responsible gamblers': a Foucauldian perspective. Inf. Commun. Technol. Law 30, 54–74. 10.1080/13600834.2020.1807117

[B3] Barry L. (2019). The rationality of the digital governmentality. J. Cult. Res. 23, 365–380. 10.1080/14797585.2020.1714878

[B4] Christiaens T. (2020). The entrepreneur of the self beyond Foucault's neoliberal homo oeconomicus. Eur. J. Soc. Theor. 23, 493–511. 10.1177/1368431019857998

[B5] Cisneros Örnberg J. Tammi T. (2011). Gambling problems as a political framing: Safeguarding the monopolies in Finland and Sweden. J. Gambl. Issues 1, 110–125. 10.4309/jgi.2011.26.8

[B6] Collier S. J. (2009). Topologies of power: Foucault's analysis of political government beyond ‘governmentality'. Theor. Cult. Soc. 26, 78–108. 10.1177/0263276409347694

[B7] Cruikshank B. (1996). “Revolutions within: self-government and self-esteem”, in *Foucault and Political Reason: Liberalism, Neo-Liberalism and Rationalities of Government*, eds. A. Barry, T. Osborne, and N. Rose (Chicago: The University of Chicago Press), 231–251.

[B8] Danske Spil (2020). Å*rsrapport 2019*. Available online at: https://danskespil.dk/-/media/files/pdf/danske-spils-aarsrapport-2019 (accessed August 18, 2022).

[B9] Danske Spil (2021). Årsrapport 2020. Available online at: https://ds-static.dk/-/media/files/pdf/arsrapport2020.ashx (accessed August 18, 2022).

[B10] Danske Spil (2022). Å*rsrapport 2021*. Available online at: https://ds-static.dk/-/media/files/pdf/danske-spil-arsrapport-2021final.ashx (accessed August 18, 2022).

[B11] Foucault M. (1978). The History of Sexuality. An Introduction. London: Penguin Books.

[B12] Foucault M. (1979). Discipline and Punish: The Birth of the Prison. New York, NY: Vintage Books.

[B13] Foucault M. (1983). “The subject and power,” in Michel Foucault: Beyond Structuralism and Hermeneutics, eds H. L. Dreyfus and P. Rabinow (Chicago: The University of Chicago Press), 208–226.

[B14] Foucault M. (1985). The History of Sexuality. The Use of Pleasure. New York, NY: Penguin.

[B15] Foucault M. (1997a). “Technologies of the self”, in Ethics: Subjectivity and Truth. Essential Works of Foucault 1954–1984, M. Foucault (New York, NY: The New Press), 224–251.

[B16] Foucault M. (1997b). “What is enlightment?”, in Ethics: Subjectivity and Truth. Essential Works of Foucault 1954–1984, M. Foucault (New York, NY: The New Press), 303–319.

[B17] Foucault M. (2007). Security, Territory, Population: Lectures at the Collège De France 1977-1978. New York, NY: Palgrave MacMillan.

[B18] Foucault M. (2008). The Birth of Biopolitics: Lectures at the Collège De France 1978-1979. Basingstoke: Palgrave Macmillan.

[B19] Foucault M. (2021). Confessions of the Flesh. The History of Sexuality. Vol. 4. New York, NY: Penguin Press.

[B20] Francis L. Livingstone C. (2021). Discourses of responsible gambling and gambling harm: observations from Victoria, Australia. Addict. Res. Theor. 29, 212–222. 10.1080/16066359.2020.1867111

[B21] Hacking I. (2002). Historical Ontology. London: Harvard University Press.

[B22] Hancock L. Smith G. (2017). Critiquing the Reno Model I-IV international influence on regulators and governments (2004–2015): the distorted reality of “responsible gambling”. Int. J. Mental Health Addict. 15, 1151–1176. 10.1007/s11469-017-9746-y

[B23] Knai C. Petticrew M. Capewell S. Cassidy R. Collin J. Cummins S.. (2021). The case for developing a cohesive systems approach to research across unhealthy commodity industries. BMJ Global Health. 6, e003543. 10.1136/bmjgh-2020-003543 33593757 PMC7888371

[B24] Lindner P. (2020). Molecular politics, wearables, and the aretaic shift in biopolitical governance. Theor. Cult. Soc. 37, 71–96. 10.1177/0263276419894053

[B25] Livingstone C. Rintoul A. (2020). Moving on from responsible gambling: a new discourse is needed to prevent and minimise harm from gambling. Pub. Health 184, 107–112. 10.1016/j.puhe.2020.03.018 32434694

[B26] Lupton D. (1995). The Imperative of Health: Public Health and the Regulated Body. London: Sage.

[B27] Lupton D. (2012). M-health and health promotion: the digital cyborg and surveillance society. Soc. Theor. Health 10, 229–244. 10.1057/sth.2012.6

[B28] Miller H. E. Thomas S. L. Smith K. M. Robinson P. (2016). Surveillance, responsibility and control: an analysis of government and industry discourses about “problem” and “responsible” gambling. Addict. Res. Theor. 24, 163–176. 10.3109/16066359.2015.1094060

[B29] Moore D. Fraser S. (2006). Putting at risk what we know: reflecting on the drug-using subject in harm reduction and its political implications. Soc. Sci. Med. 62, 3035–3047. 10.1016/j.socscimed.2005.11.067 16413645

[B30] Newall P. W. S. (2019). Dark nudges in gambling. Addict. Res. Theor. 27, 65–67. 10.1080/16066359.2018.1474206

[B31] Nikkinen J. Marionneau V. (2021). On the efficiency of Nordic state-controlled gambling companies. Nordic Stud. Alcohol Drugs 38, 212–226. 10.1177/1455072520968024 35310613 PMC8899251

[B32] Norsk Tipping (2020). Drømmer og ansvar: Norsk Tippings Års-og Samfunnsrapport 2019. Available online at: http://2019.norsk-tipping.no/wp-content/uploads/sites/2/2020/04/NorskTipping_rapport_2019_formell.pdf (accessed August 18, 2022).

[B33] Norsk Tipping (2021). Et krevende samfunnsoppdrag: Norsk Tippings Års-og Samfunnsrapport 2020. Available online at: https://2020.norsk-tipping.no/wp-content/uploads/sites/4/2021/04/NorskTipping_2020_rapport_kortversjon.pdf (accessed August 18, 2022).

[B34] Norsk Tipping (2022). Ansvarlighet, Attraktivitet og Effektiv Drift: Norsk Tippings Års-og Samfunnsrapport 2021. Available online at: https://2021.norsk-tipping.no/wp-content/uploads/sites/6/2022/04/NorskTipping_2021_rapport_kortversjon.pdf (accessed August 18, 2022).

[B35] Nowell L. S. Norris J. M. White D. E. Moules N. J. (2017). Thematic analysis: striving to meet the trustworthiness criteria. Int. J. Q. Methods 16, 1609406917733847. 10.1177/1609406917733847

[B36] O'Malley P. (1996). Risk and responsibility, in Foucault and Political Reason: Liberalism, Neo-Liberalism and Rationalities of Government. Chicago: The University of Chicago Press), 189–207.

[B37] Petersen A. R. Bunton R. (1997). Foucault, Health and Medicine. London: Routledge.

[B38] Price A. Hilbrecht M. Billi R. (2021). Charting a path towards a public health approach for gambling harm prevention. J. Pub. Health 29, 37–53. 10.1007/s10389-020-01437-2 33432287 PMC7787930

[B39] Rae G. (2022). The Ethical Self in the Later Foucault: The Question of Normativity. Bedford, NH: Sophia.

[B40] Reith G. (2004). Consumption and its discontents: addiction, identity and the problems of freedom. Br. J. Sociol. 55, 283–300. 10.1111/j.1468-4446.2004.00019.x 15233634

[B41] Reith G. (2007). Gambling and the contradictions of consumption: a genealogy of the “pathological” subject. Am. Behav. Sci. 51, 33–55. 10.1177/0002764207304856

[B42] Reith G. (2008). Reflections on responsibility. J. Gambl. Issues 22, 149–155. 10.4309/jgi.2008.22.12

[B43] Rose N. (1996). Inventing Ourselves: Psychology, Power, and Personhood. Cambridge: Cambridge University Press.

[B44] Rose N. (1999). Powers of Freedom: Reframing Political Thought. Cambridge: Cambridge University Press.

[B45] Rouvroy A. Berns T. (2013). Algorithmic governmentality and prospects of emancipation: disparateness as a precondition for individuation through relationships? Réseaux 177, 163–196. 10.3917/res.177.0163

[B46] Schüll N. D. (2006). Machines, medication, modulation: circuits of dependency and self-care in Las Vegas. Cult. Med. Psychiatr. 30, 223–247. 10.1007/s11013-006-9018-y 16944328

[B47] Selin J. (2011). Hallinnan Näkökulmia Huumeriippuvuuden Hoitoon Suomessa Vuosina 1965-2005. Dissertation, Jyväskylä: University of Jyväskylä

[B48] Selin J. (2016). From self-regulation to regulation? An analysis of gambling policy reform in Finland. Addict. Res. Theor. 24, 193–208. 10.3109/16066359.2015.1102894

[B49] Sulkunen P. Babor T. Cisneros Örnberg F. Egerer J. Hellman M. Livingstone M.. (2018). Setting Limits: Gambling, Science and Public Policy. Oxford: OUP Oxford. 10.1111/add.15241 33084199

[B50] Svenska Spel (2020). Svenska Spels Årsredovisning 2019. Available online at: https://om.svenskaspel.se/wp-content/uploads/2020/03/arsredovisningen-2019.pdf (accessed August 18, 2022).

[B51] Svenska Spel (2021). Svenska Spels Årsredovisning 2020. Available online at: https://om.svenskaspel.se/wp-content/uploads/2021/03/arsredovisningen-2020.pdf (accessed August 18, 2022).

[B52] Svenska Spel (2022). Svenska Spels Årsredovisning 2021. Available online at: https://om.svenskaspel.se/wp-content/uploads/2022/03/arsredovisningen-2021.pdf (accessed August 18, 2022).

[B53] The House of Lords (2020). Gambling harm: Time for action. Select Committee on the Social and Economic Impact of the Gambling Industry. Available online at: https://committees.parliament.uk/publications/1700/documents/16622/default/ (accessed August 22, 2022).

[B54] Tiisala T. (2017). Overcoming “the present limits of the necessary”: Foucault's conception of a critique. Southe. J. Philos. 55, 7–24. 10.1111/sjp.12224

[B55] Tiisala T. (2021). Foucault, neoliberalism, and equality. Crit. Inq. 48, 23–44. 10.1086/715986

[B56] Valverde M. (1998). Diseases of the Will: Alcohol and the Dilemmas of Freedom. Cambridge: Cambridge University Press.

[B57] Van Schalkwyk M. C. I. Petticrew M. Cassidy R. Adams P. McKee M. Reynolds J.. (2021). A public health approach to gambling regulation: Countering powerful influences. Lancet Pub. Health 6, e614–e619. 10.1016/S2468-2667(21)00098-0 34166631

[B58] Van Wijk B. (2021). Beyond the entrepreneur society: Foucault, neoliberalism and the critical attitude. Philos. Soc. Critic. 48, 1914537211017589. 10.1177/01914537211017589

[B59] Veikkaus (2020). Vuosi-ja Vastuullisuusraportti 2019. Available online at: https://cms.veikkaus.fi/site/binaries/content/assets/dokumentit/vuosikertomus/2019/veikkaus_vuosi-ja-vastuullisuusraportti_2019.pdf (accessed August 18, 2022).

[B60] Veikkaus (2021). Vuosi- a Vastuullisuusraportti 2020. Available online at: https://cms.veikkaus.fi/site/binaries/content/assets/dokumentit/vuosikertomus/2020/vuosi_-ja-vastuullisuusraportti_2020.pdf (accessed August 18, 2022).

[B61] Veikkaus (2022). Vuosi-ja Vastuullisuusraportti 2021. Available online at: https://cms.veikkaus.fi/site/binaries/content/assets/dokumentit/vuosikertomus/2021/vuosi_vastuullisuusraportti-2021.pdf (accessed August 18, 2022).

[B62] Vrecko S. (2010). ‘Civilizing technologies' and the control of deviance. BioSocieties 5, 36–51. 10.1057/biosoc.2009.8

[B63] Young M. (2013). Statistics, scapegoats and social control: a critique of pathological gambling prevalence research. Addict. Res. Theor. 21, 1–11. 10.3109/16066359.2012.680079

